# Analysis of microbial aerosols diversity in cattle farms in Ningxia

**DOI:** 10.3389/fvets.2025.1542971

**Published:** 2025-07-08

**Authors:** Yanan Guo, Yanni Mao, Shuqiang Zhao, Fei Yang, Youli Yu, Chong Chen, Mengmeng Yang, Jiandong Wang

**Affiliations:** ^1^Institute of Animal Science, Ningxia Academy of Agricultural and Forestry Sciences, Yinchuan, Ningxia, China; ^2^Guyuan Branch of Ningxia Academy of Agriculture and Forestry Sciences, Guyuan, Ningxia, China; ^3^Zhumadian Animal Disease Prevention and Quarantine Center, Zhumadian, Henan, China; ^4^Joint International Research Laboratory of Agriculture and Agri-Product Safety, Ministry of Education of China, Institutes of Agricultural Science and Technology Development, Yangzhou University, Yangzhou, China; ^5^School of Basic Medicine, Ningxia Medical University, Yinchuan, Ningxia, China

**Keywords:** bioaerosols, bacterial diversity, 16S rRNA gene sequencing, cattle barns, core microbiome

## Abstract

**Introduction:**

Beef cattle farming, a key industry in Ningxia Hui Autonomous Region, has intensified, raising public health concerns due to bioaerosol emissions. However, the distribution characteristics of these bioaerosols remain poorly understood.

**Methods:**

We characterized bacterial communities in bioaerosols from beef cattle pens across five Ningxia regions—Guyuan (G), Yinchuan (Y), Shizuishan (S), Zhongwei (Z), and Wuzhong (W)—and compared two rearing scales: smallholder farms (S) and large-scale farms (L). Using filter membrane sampling and 16S rRNA gene sequencing, we analyzed bacterial abundance and diversity.

**Results and Discussion:**

Regional differences significantly influenced bacterial abundance (*p* < 0.05), whereas rearing scales had minimal impact. We identified 45,486 operational taxonomic units (OTUs), of which 423 were shared across all samples, constituting a core microbiome that accounted for 46% of total sequences. Co-occurrence network analysis revealed greater microbial interaction complexity in regionally distinct samples. Our results elucidate the distribution of bioaerosol-borne microbial communities in cattle farms, highlighting potential transmission pathways of airborne microorganisms and informing strategies to mitigate exposure risks for livestock and workers.

## 1 Introduction

Microbial bioaerosols represent significant health hazards in modern livestock farming environments. The industrialization of animal production has increasingly relied on enclosed housing systems, where high stocking densities contribute to poor indoor air quality (1). Intensive livestock operations generate elevated concentrations of bioaerosols—airborne particles containing microorganisms (2)—making microbial community studies in barn environments a growing focus of aerobiology research (3, 4).

Current research highlights the importance of understanding bioaerosol composition and associated health impacts in livestock environments, particularly regarding occupational exposure risks in intensive farming systems (5). While early studies predominantly employed culture-based methods (6), these approaches are inherently limited by the unculturability of many microorganisms (7). More recent investigations have adopted culture-independent techniques like 16S rRNA gene sequencing to characterize livestock-associated bioaerosols (8, 9).

16S amplicon sequencing leverages the relationship between 16S rRNA genes and operational taxonomic units (OTUs) (10), circumventing cultivation requirements while enabling detection of previously uncharacterized microbes. Metagenomic DNA analysis through PCR-DGGE, quantitative PCR (qPCR), and next-generation sequencing (NGS) permits both bacterial quantification and taxonomic classification (11, 12). High-throughput sequencing not only reveals microbial diversity but also provides insights into functional potential, offering new perspectives for bioaerosol risk assessment (5).

Despite considerable research on livestock environments, significant gaps remain in understanding airborne microorganism concentrations and characteristics. The trend toward higher animal densities in modern facilities has increased bioaerosol levels, consequently elevating health risks for farm workers (3, 13). Recent reviews emphasize the need for focused research on microbial transmission mechanisms and public health impacts in high-density farming systems (5).

It is important to note that while sequencing technologies overcome cultivation biases, they present their own limitations. Result quality depends heavily on database reliability and processing protocols, including sequence alignment, annotation, and classification steps. Incomplete references or annotation errors may introduce biases in aerosol microbial diversity analyses. Our study implemented rigorous quality control measures and utilized multiple authoritative databases to minimize such potential biases.

Ningxia’s cattle industry has undergone significant development as a key agricultural region in Northwest China, with beef cattle production emerging as a major component of local economic and agricultural restructuring efforts. In response to increasing market demands for higher quality beef products, a growing number of producers are transitioning to intensive systems that incorporate several key advancements: scientific feeding protocols, enhanced disease prevention measures, optimized feed formulations, and modern facility designs including automated feeding/watering systems and advanced ventilation technologies. This industry-wide shift toward intensification, scaling, and modernization has demonstrated measurable benefits in both productivity metrics and economic returns, while simultaneously supporting broader goals of sustainable agricultural development in the Ningxia region.

This study implemented an integrated methodological framework to characterize bioaerosols within Ningxia’s transitioning agricultural landscape. We conducted systematic environmental monitoring across a representative selection of cattle facilities encompassing diverse production scales and geographical distributions throughout the region. Following this comprehensive environmental assessment, we employed 16S rRNA gene amplicon sequencing to address three core research objectives: (1) comprehensive profiling and quantitative analysis of microbial constituents in cattle barn bioaerosols, (2) in-depth characterization of taxonomic structure and spatial ecological patterns, and (3) systematic investigation of microbial interaction networks. Our integrative approach provides novel insights into the spatiotemporal composition of bioaerosol distribution in intensive cattle production systems and reveals significant correlations between microbial biodiversity and modern husbandry practices. These findings contribute substantively to the scientific basis for occupational risk assessment related to agricultural bioaerosol exposure and offer evidence-based recommendations for developing targeted intervention strategies and regulatory frameworks.

## 2 Materials and methods

### 2.1 Study design and sampling locations

This study employed a comprehensive methodological approach to characterize bioaerosols across cattle farming systems in Ningxia Hui Autonomous Region. We implemented a stratified random sampling design to select 30 cattle farms distributed across five major agricultural regions: Yinchuan (Y, 38.47°N, 106.27°E), Shizuishan (S, 39.04°N, 106.39°E), Wuzhong (W, 37.99°N, 106.21°E), Guyuan (G, 36.00°N, 106.27°E), and Zhongwei (Z, 37.50°N, 105.18°E). Farm Selection and Characteristics: Scale Stratification: Small-scale farms: <50 cattle (*n* = 15 total, 3 per region); Large-scale farms: >200 cattle (*n* = 15 total, 3 per region) (Table 1); Housing Characteristics: naturally ventilated barns with concrete floors; comparable construction materials across all facilities; Small-scale: 5–7 m^2^/animal, Large-scale: 3–5 m^2^/animal, animal Management: standardized vaccination protocols, silage-based feeding regimens, beef cattle aged 12–24 months, no calves or lactating cows present. Bioaerosol Collection Protocol: sampling was conducted during autumn (September–November 2023) to minimize seasonal variability. At each farm: Collected six aerosol size fractions (0.65– > 7.0 μm) simultaneously; performed duplicate measurements for each size fraction (total 12 samples/farm); sampling height: 1.5 m (breathing zone); sampling time: 08:00–10:00 (peak activity period); duration: 5–10 min (adjusted based on particle loading). Each farm contributed 12 aerosol samples (6 size fractions × 2 replicates), totaling 720 initial collections. For microbial community analysis, size fractions from each farm were pooled proportionally by particle concentration after DNA extraction, yielding 60 composite samples (30 farms × 2 scales) for sequencing. This pooling strategy balanced resolution of size-specific patterns with practical sequencing constraints.

**Table 1:** Sampling design summary.

Region	Small-scale farms (*n*)	Large-scale farms (*n*)	Total samples
Y	3	3	12
S	3	3	12
W	3	3	12
G	3	3	12
Z	3	3	12
Total	15	15	60

### 2.2 Bioaerosol DNA extraction and genome library preparation

DNA was extracted separately from each of the 720 individual size-fraction samples. Post-extraction, equal masses of DNA from the six size fractions per farm were pooled prior to library preparation, maintaining separate pools for each replicate (total 60 libraries: 30 farms × 2 replicates). Negative controls (*n* = 5) were processed identically to monitor cross-contamination. Total genomic DNA was extracted from each of the 60 aerosol size-fraction samples (processed separately) using the E.Z.N.A.^®^ Soil DNA Kit (Omega Bio-tek, Norcross, GA, United States), with the following modifications: (1) extended bead-beating duration (5 min), (2) supplemental proteinase K digestion step, and (3) inclusion of negative extraction controls (*n* = 5) and positive controls (ZymoBIOMICS Microbial Community Standard). DNA quality was rigorously assessed, demonstrating A260/280 ratios >1.8 and concentrations exceeding 20 ng/μL, as verified by NanoDrop ND-1000 spectrophotometry (Thermo Scientific, Wilmington, DE, United States) and Qubit 4.0 Fluorometric quantification (Thermo Fisher Scientific, Waltham, MA, United States).

The V3–V4 hypervariable region of bacterial 16S rRNA genes was amplified using barcoded primers 338F (5’-ACTCCTACGGGAGGCAGCAG-3′) and 806R (5’-GGACTACHVGGGTWTCTAAT-3′) under optimized thermal cycling conditions: initial denaturation at 95°C for 3 min; 30 cycles of 95°C for 30s, 55°C for 30s, and 72°C for 45 s; followed by final extension at 72°C for 10 min. Purified amplicons were subjected to paired-end sequencing (2 × 300 bp) on the Illumina MiSeq platform (Illumina Inc., San Diego, CA, United States), achieving a target depth of 50,000 read-pairs per sample (actual range: 45,217–52,103).

### 2.3 Sequence data processing

Sequence data processing was performed using an optimized bioinformatics pipeline on the Majorbio Cloud Platform. Raw sequencing reads underwent quality filtering and denoising through DADA2 implemented in QIIME2 (v2021.11), with specific trimming parameters (trim-left-f 17, trim-left-r 21; trunc-len-f 280, trunc-len-r 220) to ensure read quality. Chimeric sequences were subsequently removed using VSEARCH, followed by taxonomic classification against the SILVA v138 reference database with a minimum bootstrap confidence threshold of 80%. To ensure data comparability, samples were rarefied to 45,000 sequences per sample after removing singleton ASVs. Potential contaminants identified in negative controls (average 1,243 reads/sample) were systematically removed using the decontam package (prevalence method, threshold = 0.1), ensuring the reliability of downstream analyses.

### 2.4 Statistical analysis

Microbial diversity assessment employed a multi-tiered analytical approach implemented in R (v4.2.0). Alpha diversity was evaluated using three complementary metrics: observed ASV richness, Shannon diversity index, and Faith’s phylogenetic diversity, with group comparisons conducted using non-parametric Kruskal-Wallis tests followed by Dunn’s post-hoc comparisons for significant results. Beta diversity patterns were visualized through principal coordinates analysis (PCoA) of Bray–Curtis dissimilarity matrices, with statistical significance assessed via PERMANOVA (adonis2 function, 999 permutations) after verifying homogeneity of dispersion using betadisper tests. Differential abundance analysis was performed using linear discriminant analysis effect size (LEfSe) with stringent thresholds (LDA score >2, Benjamini-Hochberg corrected q < 0.05). Microbial co-occurrence networks were constructed through SparCC correlation analysis (absolute *ρ* > 0.6, *p* < 0.01) and visualized in Gephi v0.9.2, with edge weights reflecting correlation strength. All models accounted for geographical region and farming scale as stratification variables to isolate their independent effects.

## 3 Results

### 3.1 Bacterial diversity in cattle barn bioaerosols

Microbiological analysis of cattle barn bioaerosols revealed significant spatial and operational patterns in bacterial community composition. Sequencing efforts yielded an average of 4,021,916 raw reads per sample, with 3,862,616 high-quality reads retained after quality control (96.04% processing efficiency). At a 97% sequence similarity threshold, these reads clustered into 45,486 operational taxonomic units (OTUs), demonstrating substantial microbial diversity within the sampled environments. Rarefaction analysis based on the ASV richness confirmed adequate sampling depth across all collections (Supplementary Figure S1).

**Figure 1:**
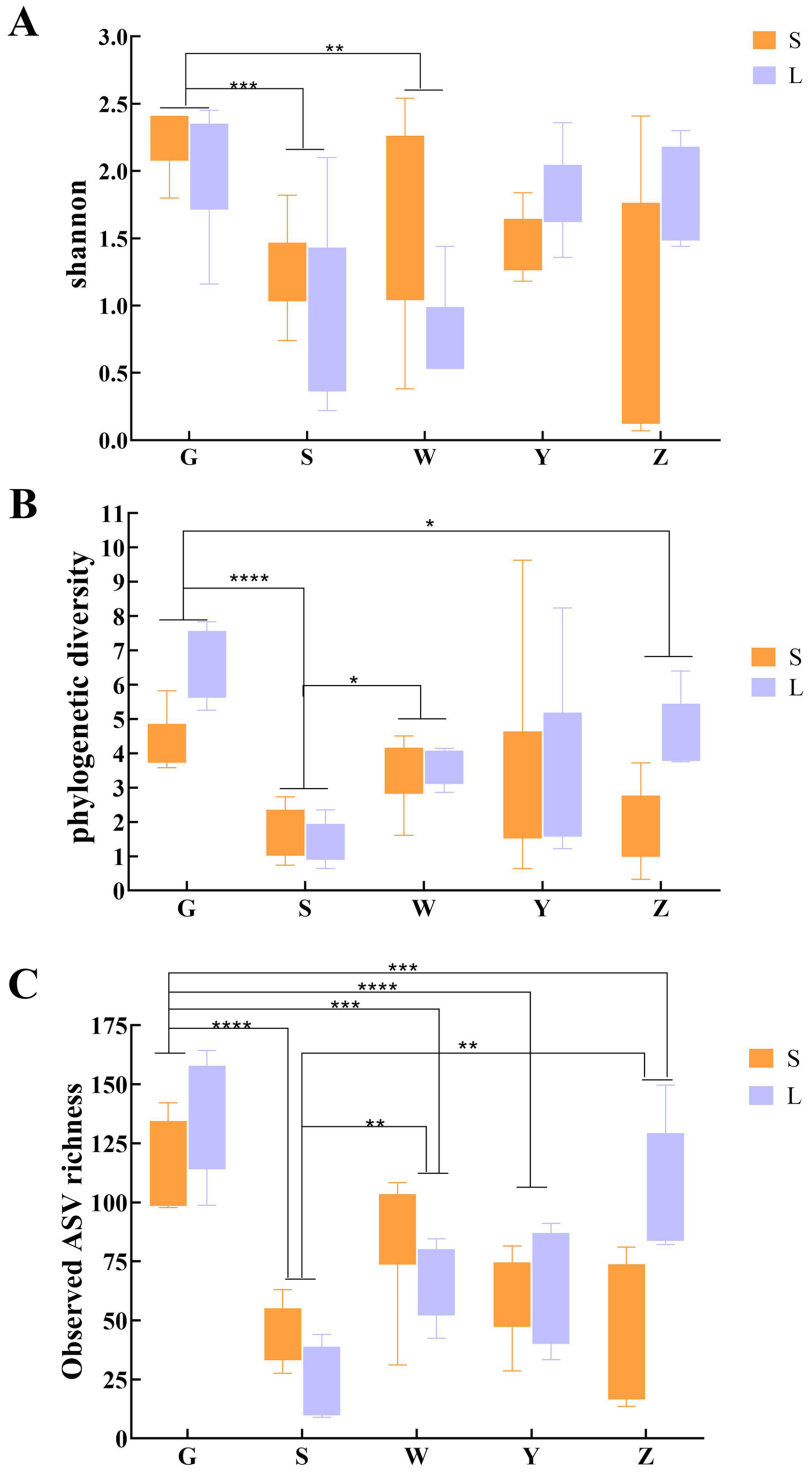
Alpha diversity comparisons of bacterial communities in air samples from cattle barns based on Shannon **(A)**, PD **(B)**, and observed values **(C)**.

**Figure 2:**
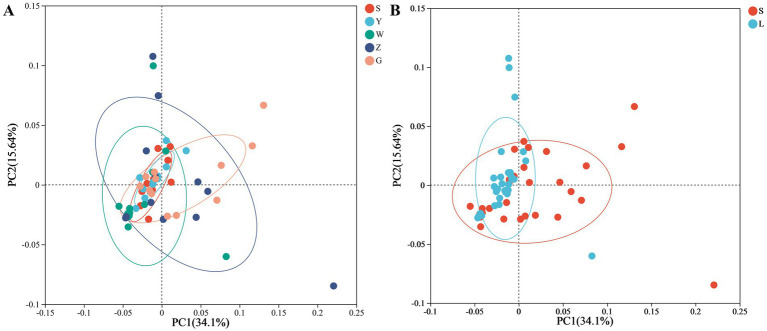
Microbial community structure in cattle barn air samples. **(A)** Principal coordinates analysis (PCoA) of Bray–Curtis dissimilarity showing regional differences (34.1% variation explained, PERMANOVA *p* < 0.001). **(B)** PCoA showing farming scale differences (small-scale vs. large-scale; 34.1% variation explained, PERMANOVA *p* < 0.05). Ellipses represent 95% confidence intervals. Analyses controlled for homogeneity of dispersion (betadisper, *p* > 0.1). Points are colored by region **(A)** or farming scale **(B)**, with shape indicating the other factor.

Regional comparisons revealed significant disparities in microbial diversity metrics (Figure 1). Bioaerosols from Guyuan (G) demonstrated consistently higher diversity than other regions, with statistically significant increases of: 28.7% in Shannon index (*p* < 0.01, Kruskal-Wallis with Dunn’s post-hoc), 34.2% in Phylogenetic Diversity (PD; *p* < 0.005). Notably, small-scale farms (S) exhibited greater within-region variability in PD compared to large-scale farms (L), particularly in Wuzhong (W) and Zhongwei (Z). Pairwise comparisons showed: G-S vs. G-L: No significant difference (*p* = 0.12), Y-S vs. Y-L: 22% lower PD in Y-L (*p* < 0.05). Negative control analysis identified 187 ASVs (0.41% of total) through the decontam pipeline, representing 1.2 ± 0.4% (range: 0.7–2.1%) of reads per sample. These potential contaminants were predominantly classified as: *Pseudomonas* (23.5% of contaminant ASVs), *Staphylococcus* (18.7%), *Laboratory reagents* (16.3% unclassified Bacteria). All contaminant ASVs were removed prior to diversity analyses, with sample-specific abundance losses documented in Supplementary Figure S2. Beta diversity assessment through Bray–Curtis dissimilarity and principal coordinate analysis (PCoA) quantified these regional effects, revealing that geographical location accounted for 34.1% of observed community variation (PERMANOVA, *p* < 0.001). While the PCoA plot showed moderate visual clustering, the statistical significance indicates that underlying environmental parameters-including climatic conditions and regional husbandry practices-substantially influence bioaerosol microbiome composition (Figure 2). PERMANOVA of the full multivariable model (region + scale + interaction) explained 42.4% of community variation (pseudo-*F* = 8.31, *p* = 0.001), with: Region: R^2^ = 34.1% (*p* = 0.001), Scale: R^2^ = 8.3% (*p* = 0.01), Interaction: R^2^ = 3.7% (*p* = 0.12). Beta-dispersion tests confirmed homogeneity of variances (*p* > 0.05 for all factors), validating PERMANOVA assumptions.

In contrast to the strong regional effects, farming scale demonstrated more modest impacts on microbial communities, explaining only 8.3% of compositional variation (PERMANOVA, *p* < 0.05). The PCoA visualization suggested these scale-related differences primarily distinguished smallholder operations from commercial facilities, likely reflecting disparities in environmental management practices. The magnitude of scale effects showed regional variation, indicating interactive effects between geographical context and farm management approaches in shaping bioaerosol microbiomes. These findings highlight the complex interplay of spatial and operational factors in determining agricultural bioaerosol characteristics, with important implications for understanding potential exposure risks in different farming contexts.

### 3.2 Microbial compositional differences in cattle barn bioaerosols

**Figure 3:**
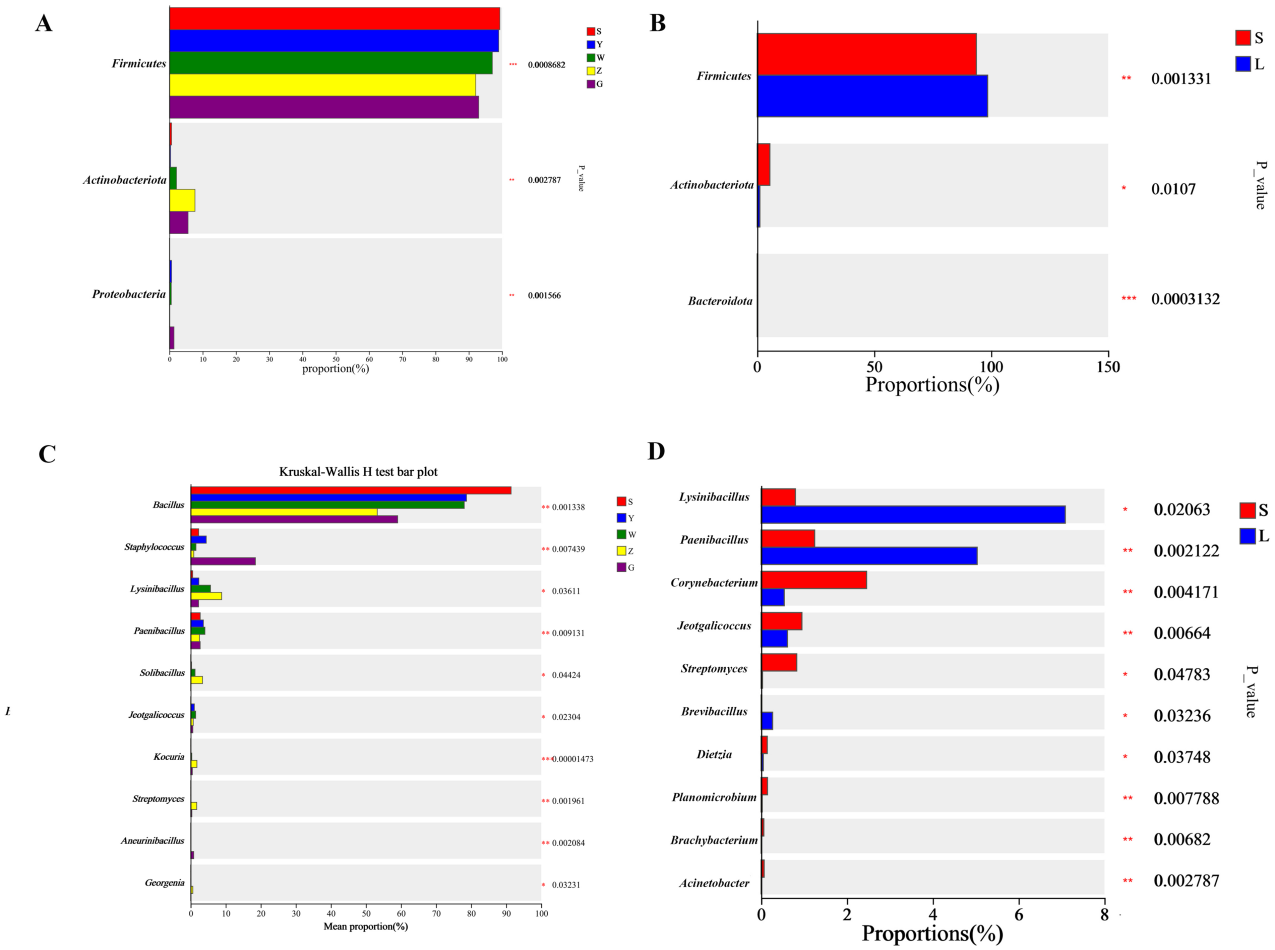
Differentially abundant bacterial taxa in cattle barn bioaerosols. **(A,B)** Phylum and **(C,D)** genus-level relative abundances across regions **(A,C)** and farming scales **(B,D)**. Dots represent mean relative abundance (log10 scale). Only taxa with significant differences (Kruskal-Wallis with Benjamini-Hochberg correction, q < 0.05) are shown. Error bars show standard error.

Taxonomic analysis revealed significant microbial profile variations across Ningxia’s cattle barns (Figure 3). *Firmicutes* and *Actinobacteriota* emerged as the dominant phyla, collectively representing 62–78% of relative abundance across all samples, with *Firmicutes* particularly abundant in large-scale farms (38.5–42.1%) compared to small-scale operations (Supplementary Figures S2, S3). At the regional level, Shizuishan and Yinchuan exhibited significantly higher *Firmicutes* abundance (34–38%) relative to other regions (28.4 ± 2.3%, q < 0.05), while Zhongwei demonstrated the highest *Actinobacteriota* levels (22.5 ± 1.9% vs. regional average 17.3 ± 1.4%, q < 0.05). Farming scale substantially influenced microbial composition, with large-scale operations showing 18.7% greater *Firmicutes* abundance (q < 0.05) and 2.3-fold higher *Lysinibacillus* counts (q < 0.01), whereas small-scale farms maintained elevated populations of *Streptomyces* (3.1 ± 0.3% vs. 1.8 ± 0.2%, q < 0.05) and *Corynebacterium* species. Genus-level analysis (Kruskal-Wallis q < 0.05) showed: *Bacillus* predominated in Shizuishan (9.4 ± 0.8%) and Yinchuan (8.1 ± 0.7%), *Lysinibacillus* marked Zhongwei samples (5.2 ± 0.4%), and *Corynebacterium* characterized Guyuan (5.9 ± 0.5%). These compositional differences correlated strongly with measured environmental parameters and management practices, providing mechanistic insights into the observed biogeographic patterns.

### 3.3 Study of bacterial diversity in air samples from cattle barns

Microbial community analysis revealed both conserved and distinctive elements across geographical and operational scales. The study identified a substantial core microbiome, with 20.62% (423 of 2,051) of bacterial genera being shared across all five sampled regions (Supplementary Figure S4A). This conserved component represented the dominant fraction of sequence reads (68.3 ± 5.2% of total abundance), indicating the establishment of stable microbial populations in cattle barn environments regardless of location.

Regional specialization was evident through distinct distribution patterns: Guyuan exhibited the highest proportion of unique genera (15.88%), followed by Zhongwei (6.64%) and Yinchuan (4.98%); Wuzhong and Shizuishan showed relatively lower endemicity (4.03 and 1.66% unique genera respectively); Region-specific taxa typically occurred in lower abundance (0.5–3.2% relative abundance) compared to core microbiome members. Comparative analysis between farming scales demonstrated: 56.16% genera overlap between smallholder (S) and large-scale (L) operations; Smallholder farms harbored 2.3-fold more unique genera (30.18%) than large-scale facilities (13.03%); Scale-specific taxa showed differential abundance patterns (Supplementary Figure S4B): Smallholder-associated genera: 1.8–4.7% relative abundance; Large-scale-associated genera: 0.9–3.5% relative abundance. These findings support an ecological model where cattle barn bioaerosols maintain: A stable core microbiome adapted to the cattle rearing environment; Regionally distinct microbial signatures influenced by local climatic and management factors; Scale-dependent variations reflecting differences in housing density and operational practices. The predominance of shared microbial taxa suggests strong environmental selection pressures in cattle production systems, while the presence of niche-specific populations indicates opportunities for targeted microbial management based on regional and operational characteristics.

### 3.4 Representative genera in the core microbiome of air samples from cattle barns

Analysis of the aerosol microbiome revealed a remarkably conserved core community despite the high overall microbial diversity observed. From the total 45,486 operational taxonomic units (OTUs) identified across all samples, only 423 OTUs (0.93%) were shared universally, yet these core taxa accounted for a substantial 46% of total sequence reads, demonstrating their numerical dominance in the bioaerosol communities. As indicated in Table 2, taxonomic characterization showed this core microbiome spanned seven bacterial phyla, with *Firmicutes* representing the most substantial component (148 OTUs, 35.0% of core taxa), followed by *Actinobacteriota* (99 OTUs, 23.4%) and *Proteobacteria* (41 OTUs, 9.7%). Less abundant but consistently present phyla included *Bacteroidota* (7 OTUs), unclassified denitrifying bacteria (22 OTUs), *Campylobacterota* (4 OTUs), and a single representative of candidate phylum *Patescibacteria*. Regional analysis confirmed the ubiquity of this core community, with its members consistently representing over one-third of sequence reads in all geographical locations (range: 38–52%, mean 46%). The disproportionate representation of these core OTUs, particularly within the *Firmicutes* and *Actinobacteriota phyla*, suggests these microbial groups have evolved specialized adaptations to the cattle barn aerosol environment, maintaining stable populations despite regional variations in climate and management practices. This finding highlights the existence of a highly selected, environmentally resilient microbial consortium that forms the foundational population of cattle barn bioaerosols across diverse geographical and operational contexts in Ningxia.

**Table 2:** Core microbiome composition across regions.

Area	Bacteria phylum level	OTUs
Guyuan	Actinobacteria phylum	291
	Unclassified phylum denitrifying bacteria	5
	Anaplasma phylum	294
	Actinobacteria of the phylum Thick-walled	45
	Actinobacteria	1
Yinchuan	Actinobacteria phylum	219
	Unclassified phylum denitrifying bacteria	2
	Anaplasma phylum	8
	Actinobacteria of the phylum Thick-walled	58
	Actinobacteria	0
Wuzhong	Actinobacteria phylum	254
	Unclassified phylum denitrifying bacteria	4
	Anaplasma phylum	257
	Actinobacteria of the phylum Thick-walled	44
	Actinobacteria	97
Shizuishan	Actinobacteria phylum	157
	Actinobacteria	62
	Actinobacteria of the phylum Thick-walled	58
	Actinobacteria	58
	Unclassified phylum denitrifying bacteria	58
Zhongwei	Actinobacteria phylum	965
	Unclassified phylum denitrifying bacteria	20
	Anaplasma phylum	58
	Actinobacteria of the phylum Thick-walled	62
	Actinobacteria	42

Summary table showing phylum and genus-level taxa present in ≥90% samples from each region, with mean relative abundance (%) and prevalence (%).

### 3.5 Co-occurrence networks of microbial communities in air samples from cattle barns

**Figure 4:**
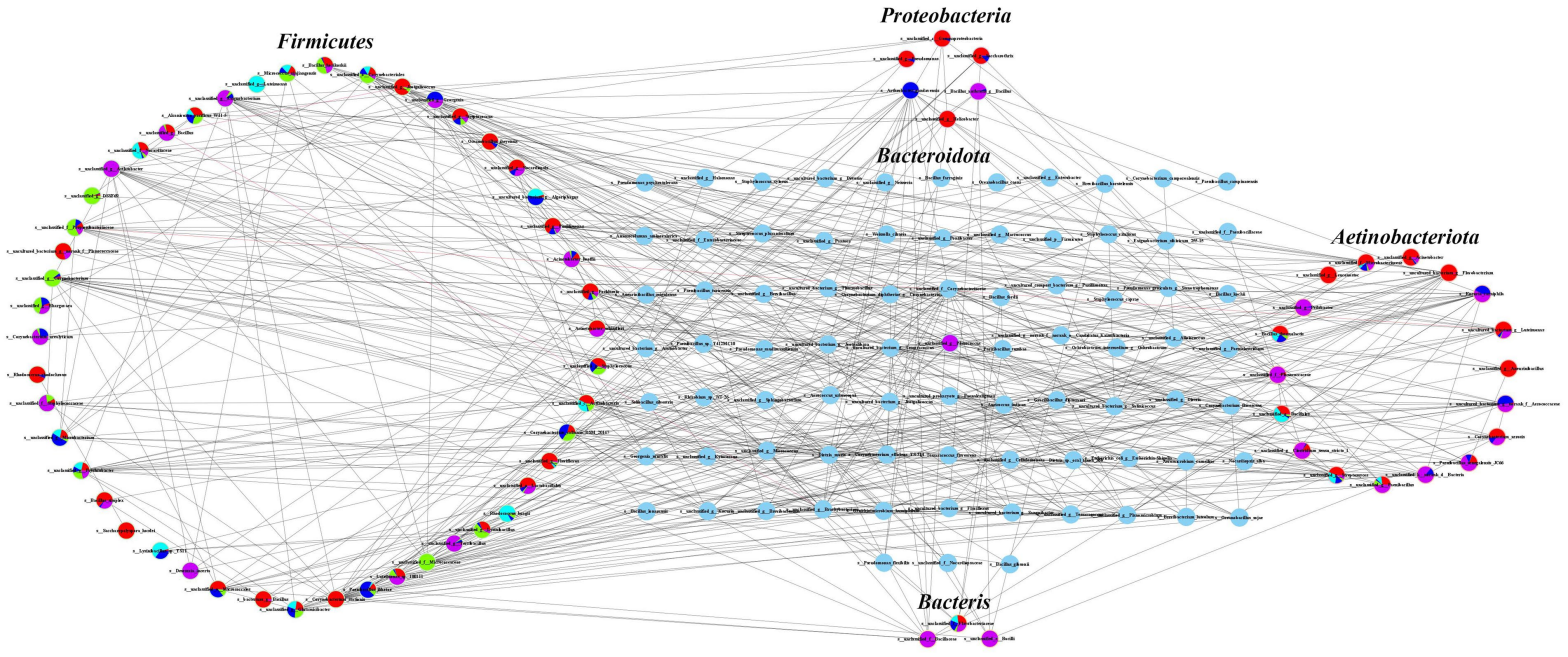
Symbiotic network analysis of microbial communities in air samples from beef cattle breeding pens.

Co-occurrence network analysis revealed complex ecological correlations within the airborne microbial communities across different geographical regions and farming scales. The SparCC (14) correlation analysis (*p* < 0.05, |*ρ*| > 0.3) identified distinct network patterns among dominant bacterial phyla, with *Firmicutes* demonstrating the most extensive interaction network comprising 48 nodes and 148 significant edges (Figure 4). Within this phylum, the unclassified Bacillus genus exhibited notable negative correlations with *Brachybacterium* and *Jeotgalicoccus*, suggesting potential competitive exclusion composition. *Actinobacteriota* formed a moderately complex network with 18 nodes and 100 correlation edges, while *Proteobacteria* and *Bacteroidota* showed relatively simpler interaction patterns with 6 nodes (40 edges) and 7 nodes (80 edges) respectively. The unclassified denitrifying bacteria displayed the most limited network, containing only 3 nodes with 22 significant correlations.

Regional analysis uncovered distinct topological patterns in microbial networks, with Guyuan and Wuzhong samples exhibiting particularly complex correlations among *Actinobacteriota*, *Firmicutes*, and *Proteobacteria*. Shizuishan samples showed strong interconnectivity between *Actinobacteriota* and *Firmicutes*, while Yinchuan samples were dominated by *Firmicutes*-associated correlations. Zhongwei displayed the most balanced network structure, with significant correlations observed across all five major phyla. These network variations likely reflect adaptations to regional environmental conditions and management practices, with more complex interaction patterns emerging in areas with greater climatic variability. The observed network structures provide critical insights into microbial community stability and potential microbial transmission pathways, highlighting how regional factors may influence both ecological composition and health risks associated with cattle barn bioaerosols.

## 4 Discussion

The intensification of livestock production has raised significant concerns regarding indoor air quality in animal housing facilities (15), with bioaerosol emissions representing a potential public health challenge (16, 17). Our study provides novel insights into the microbial composition of cattle barn bioaerosols, addressing a critical knowledge gap in beef cattle production systems. Modern high-density farming operations generate substantial bioaerosol loads, with previous studies reporting airborne bacterial concentrations reaching 10^5^ CFU/m^3^ (18, 19). The seasonal variation observed in these concentrations, particularly the reduction during summer months due to increased ventilation (20), underscores the importance of environmental management in controlling bioaerosol dispersion.

Recent advances in molecular techniques have revolutionized our understanding of bioaerosol microbiota. While traditional culture methods have been valuable for quantifying viable bacteria, contemporary studies demonstrate that sequencing-based approaches reveal microbial diversity that is approximately three orders of magnitude greater than culture-dependent assessments (21). This technological advancement is particularly significant given the growing recognition that culture methods may systematically underestimate specific bacterial groups in aerosols (22, 23). Our findings align with these observations, confirming the superior capability of high-throughput sequencing in characterizing complex airborne microbial communities.

The microbial profiles observed in our study exhibited both universal patterns and system-specific characteristics. *Firmicutes* and *Actinobacteriota* emerged as the dominant phyla across all sampling sites, consistent with findings from other agricultural and built environments (24, 25). However, the regional variations in their relative abundance and the distinct genus-level patterns we identified provide new evidence for the influence of local environmental conditions and management practices on bioaerosol composition. Notably, the Bray–Curtis dissimilarity analysis demonstrated clear differentiation of microbial communities by both geographical location and farming scale, suggesting that these factors interact to shape the aerosol microbiome.

The ecological significance of our findings extends beyond microbial taxonomy. The identification of a core microbiome comprising only 0.93% of total OTUs yet accounting for 46% of sequence reads suggests strong environmental selection pressures in cattle barn ecosystems. This core community, dominated by *Firmicutes* and *Actinobacteriota*, likely represents microbial lineages with specialized adaptations to the aerosolized state and the barn environment (26). The co-occurrence network analysis further revealed complex ecological correlations within these communities, with distinct network topologies emerging in different regions, potentially reflecting adaptations to local environmental conditions.

From a public health perspective, our results highlight the need for region-specific risk assessments and mitigation strategies. The regional variations in microbial composition and interaction networks may translate to differences in exposure risks for farm workers and nearby communities (8, 27). Furthermore, the identification of scale-dependent microbial patterns suggests that intensification of cattle production could alter bioaerosol characteristics in predictable ways, with potential implications for occupational health management.

While this study provides important baseline data, several questions remain for future investigation. The functional attributes of the core microbiome members, their survival mechanisms in aerosols, and their potential roles in microbial transmission warrant further exploration. Additionally, longitudinal studies tracking seasonal variations in bioaerosol composition would enhance our understanding of the temporal composition in these systems. The development of standardized protocols for bioaerosol sampling and analysis in agricultural settings would facilitate more robust comparisons across studies and production systems. This study underlines the importance of characterizing bioaerosol composition in beef cattle operations, particularly for identifying potential zoonotic microbial s that may impact both animal and human health. Future research should focus on: (1) targeted detection of known microbial s, (2) evaluating transmission risks, and (3) developing evidence-based intervention strategies to mitigate exposure hazards.

## 5 Conclusion

This study investigated bacterial diversity in bioaerosols from beef cattle barns of different scales across five regions in Ningxia. The findings reveal distinct characteristics of bacterial diversity in cattle barn air, shaped by regional and scale-related differences, providing novel insights into the microbial ecology of cattle barn environments. This research enhances understanding of microbial communities in bioaerosols and establishes a foundation for evaluating the health risks associated with cattle barn environments.

## Data Availability

The original contributions presented in the study are included in the article/Supplementary material, further inquiries can be directed to the corresponding authors.

## Supplementary material

SUPPLEMENTARY FIGURE S1 Rarefaction curves for the observed values.

SUPPLEMENTARY FIGURE S2 Dominant bacterial taxa composition. **(A)** Phylum-level relative abundance across regions (small-scale farms). **(B)** Phylum-level relative abundance across regions (large-scale farms). **(C)** Genus-level relative abundance across regions (small-scale farms). **(D)** Genus-level relative abundance across regions (large-scale farms). Only taxa with >1% relative abundance in any sample are shown. Error bars represent standard error of the mean (SEM).

SUPPLEMENTARY FIGURE S3 Heatmap visualization of microbial communities. **(A)** Phylum and **(B)** Genus-level relative abundances (log10-transformed) across all samples. Columns represent individual samples grouped by region (color bars) and farming scale (small-scale/large-scale). Only taxa present at >0.1% relative abundance in at least 10% of samples are shown.

SUPPLEMENTARY FIGURE S4 Shared and unique bacterial genera across groups. Venn diagrams showing overlap of bacterial genera (rarefied to 45,000 sequences/sample) between **(A)** regions (core microbiome = 423 shared genera) and **(B)** farming scales (small-scale vs large-scale). Numbers indicate count of unique/shared genera at ≥0.01% relative abundance. Diagrams were generated using rarefied ASV tables to ensure equal sampling depth.

## References

[1] Schauberger G Mikovits C Zollitsch W Hörtenhuber SJ Baumgartner J Niebuhr K. Global warming impact on confined livestock in buildings: efficacy of adaptation measures to reduce heat stress for growing-fattening pigs. Clim Chang. (2019) 156:567–87. doi: 10.1007/s10584-019-02525-3

[2] Yousefzadeh A Maleki A Athar SD Darvishi E Ahmadi M Mohammadi E. Evaluation of bio-aerosols type, density, and modeling of dispersion in inside and outside of different wards of educational hospital. Environ Sci Pollut Res Int. (2022) 29:14143–57. doi: 10.1007/s11356-021-16733-x, PMID: 34601681 PMC8487404

[3] Douglas P Robertson S Gay R Hansell AL Gant TW. A systematic review of the public health risks of bioaerosols from intensive farming. Int J Hyg Environ Health. (2018) 221:134–73. doi: 10.1016/j.ijheh.2017.10.019, PMID: 29133137

[4] Yoo K Lee TK Choi EJ Yang J Shukla SK Hwang SI. Molecular approaches for the detection and monitoring of microbial communities in bioaerosols: a review. J Environ Sci (China). (2017) 51:234–47. doi: 10.1016/j.jes.2016.07.002, PMID: 28115135

[5] Yu G Wang X Song Z Cai Y. A review on microbial aerosols in livestock and poultry environments: pollution characteristics, damage mechanisms, and mitigation measures. Front Environ Sci Eng. (2024) 18:150. doi: 10.1007/s11783-024-1910-6

[6] Boifot KO Gohli J Moen LV Dybwad M. Performance evaluation of a new custom, multi-component DNA isolation method optimized for use in shotgun metagenomic sequencing-based aerosol microbiome research. Environ Microbiome. (2020) 15:1. doi: 10.1186/s40793-019-0349-z, PMID: 33902731 PMC8067373

[7] Kumari P Choi HL. Manure removal system influences the abundance and composition of airborne biotic contaminants in swine confinement buildings. Environ Monit Assess. (2015) 187:537. doi: 10.1007/s10661-015-4759-0, PMID: 26220780

[8] Hong PY Li X Yang X Shinkai T Zhang Y Wang X. Monitoring airborne biotic contaminants in the indoor environment of pig and poultry confinement buildings. Environ Microbiol. (2012) 14:1420–31. doi: 10.1111/j.1462-2920.2012.02726.x, PMID: 22414212

[9] Kristiansen A Saunders AM Hansen AA Nielsen PH Nielsen JL. Community structure of bacteria and fungi in aerosols of a pig confinement building. FEMS Microbiol Ecol. (2012) 80:390–401. doi: 10.1111/j.1574-6941.2012.01305.x, PMID: 22242889

[10] Poretsky R Rodriguez-R LM Luo C Tsementzi D Konstantinidis KT. Strengths and limitations of 16S rRNA gene amplicon sequencing in revealing temporal microbial community composition. PLoS One. (2014) 9:e93827. doi: 10.1371/journal.pone.0093827 24714158 PMC3979728

[11] White JK Nielsen JL Madsen AM. Microbial species and biodiversity in settling dust within and between pig farms. Environ Res. (2019) 171:558–67. doi: 10.1016/j.envres.2019.01.008, PMID: 30771719

[12] Jian C Luukkonen P Yki-Jarvinen H Salonen A Korpela K. Quantitative PCR provides a simple and accessible method for quantitative microbiota profiling. PLoS One. (2020) 15:e0227285. doi: 10.1371/journal.pone.0227285, PMID: 31940382 PMC6961887

[13] Zhao Y Aarnink A De Jong M Koerkamp G. Airborne microorganisms from livestock production systems and their relation to dust. Crit Rev Environ Sci Technol. (2014) 44:1071–128. doi: 10.1080/10643389.2012.746064 32288664 PMC7113898

[14] Friedman J Alm EJ. Inferring correlation networks from genomic survey data. PLoS Comput Biol. (2012) 8:e1002687. doi: 10.1371/journal.pcbi.1002687, PMID: 23028285 PMC3447976

[15] Zhang J Liu C Zhao G Li M Ma D Meng Q. PM2.5 synergizes with *Pseudomonas aeruginosa* to suppress alveolar macrophage function in mice through the mTOR pathway. Front Pharmacol. (2022) 13:924242. doi: 10.3389/fphar.2022.924242, PMID: 35800443 PMC9253536

[16] Cho YS Hong SC Choi J Jung JH. Development of an automated wet-cyclone system for rapid, continuous and enriched bioaerosol sampling and its application to real-time detection. Sens Actuators B Chem. (2019) 284:525–33. doi: 10.1016/j.snb.2018.12.155, PMID: 32288254 PMC7111469

[17] Wang C Lu S Zhang Z. Inactivation of airborne bacteria using different UV sources: performance modeling, energy utilization, and endotoxin degradation. Sci Total Environ. (2019) 655:787–95. doi: 10.1016/j.scitotenv.2018.11.266, PMID: 30481706 PMC7112078

[18] Bonifait L Veillette M Letourneau V Grenier D Duchaine C. Detection of *Streptococcus suis* in bioaerosols of swine confinement buildings. Appl Environ Microbiol. (2014) 80:3296–304. doi: 10.1128/AEM.04167-13, PMID: 24632262 PMC4018844

[19] Blais-Lecours P Perrott P Duchaine C. Non-culturable bioaerosols in indoor settings: impact on health and molecular approaches for detection. Atmos Environ. (2015) 110:45–53. doi: 10.1016/j.atmosenv.2015.03.039, PMID: 32288547 PMC7108366

[20] Duchaine C Grimard Y Cormier Y. Influence of building maintenance, environmental factors, and seasons on airborne contaminants of swine confinement buildings. Am Ind Hyg Assoc J. (2000) 61:56–63. doi: 10.1080/15298660008984515, PMID: 10772615

[21] Nehme B Letourneau V Forster RJ Veillette M Duchaine C. Culture-independent approach of the bacterial bioaerosol diversity in the standard swine confinement buildings, and assessment of the seasonal effect. Environ Microbiol. (2008) 10:665–75. doi: 10.1111/j.1462-2920.2007.01489.x, PMID: 18237302

[22] Pausan MR Blohs M Mahnert A Moissl-Eichinger C. The sanitary indoor environment-a potential source for intact human-associated anaerobes. NPJ Biofilms Microbiomes. (2022) 8:44. doi: 10.1038/s41522-022-00305-z, PMID: 35650275 PMC9160270

[23] Sultana S Khan MN Hossain MS Dai J Rahman MS Salimullah M. Community structure and functional annotations of the skin microbiome in healthy and diseased catfish, *Heteropneustes fossilis*. Front Microbiol. (2022) 13:856014. doi: 10.3389/fmicb.2022.856014 35295300 PMC8918984

[24] Dong B Lin X Jing X Hu T Zhou J Chen J. A bacterial genome and culture collection of gut microbial in weanling piglet. Microbiol Spectr. (2022) 10:e0241721. doi: 10.1128/spectrum.02417-21, PMID: 35171009 PMC8849097

[25] Hsu B Chen J Hsu G Hsu BM Chen JS Hsu GJ. Role of bioaerosols on the short-distance transmission of multidrug-resistant methicillin-resistant *Staphylococcus aureus* (MRSA) in a chicken farm environment. Antibiotics. (2022) 11:81. doi: 10.3390/antibiotics11010081, PMID: 35052958 PMC8773248

[26] Kraemer JG Aebi S Oppliger A Hilty M. The indoor-air microbiota of pig farms drives the composition of the pig farmers' nasal microbiota in a season-dependent and farm-specific manner. Appl Environ Microbiol. (2019) 85:e03038-18. doi: 10.1128/AEM.03038-18, PMID: 30824439 PMC6495764

[27] Gao XL Shao MF Wang Q Wang LT Fang WY Ouyang F. Airborne microbial communities in the atmospheric environment of urban hospitals in China. J Hazard Mater. (2018) 349:10–7. doi: 10.1016/j.jhazmat.2018.01.043, PMID: 29414740

