# Supplementary material for: Analysis of microbial aerosols diversity in cattle farms in Ningxia
Source: Front Vet Sci. 2025 Jul 8;12:1542971. doi: 10.3389/fvets.2025.1542971 (PMC12279523; doi:10.3389/fvets.2025.1542971)
Supplement: SUPPLEMENTARY FIGURE S1 — Rarefaction curves for the observed values. [file Data_Sheet_1.DOCX]

***Supplementary Material***

Analysis of Microbial Aerosols Diversity in Cattle Farms in Ningxia

Yanan Guo^1†^,Yanni mao^2†^,Shuqiang Zhao^3^,Fei Yang^1^,Youli Yu^1^,Chong Chen^4^,Mengmeng Yang^5*^,Jiandong Wang^1*^

^1^ Institute of Animal Science, Ningxia Academy of Agricultural and Forestry Sciences, Yinchuan, Ningxia, 750002, China

^2^ Guyuan Branch of Ning Xia Academy of Agriculture and Forestry Sciences, Guyuan, Ningxia, 756000, China

^3^ Zhumadian Animal Disease Prevention and Quarantine Center, Zhumadian, Henan, 463000, China

^4^ Joint International Research Laboratory of Agriculture and Agri-Product Safety, Ministry of Education of China, Institutes of Agricultural Science and Technology Development, Yangzhou University, Yangzhou, 225000, China

^5^ School of Basic Medicine, Ningxia Medical University, Yinchuan, Ningxia 750001, China

*** Correspondence:**
1. Mengmeng Yang

[15809605110@163.com](mailto:15809605110@163.com)

2. Jiandong Wang

[jiandongwang668@126.com](mailto:jiandongwang668@126.com)

†These authors contributed equally.

# Supplementary Figures

**
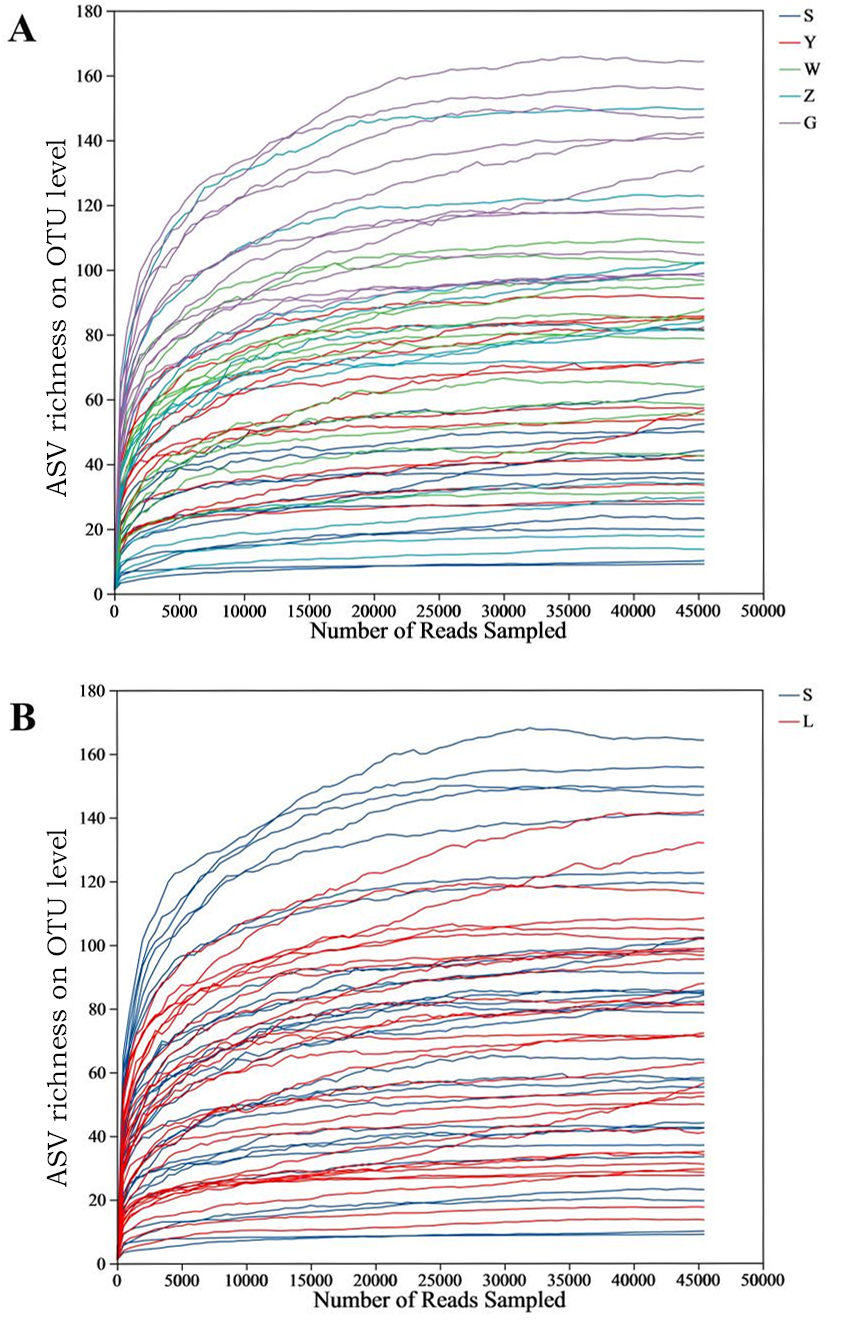
**

**Figure S1. Rarefaction analysis of sequencing depth**

Rarefaction curves showing (A) Observed ASV richness, (B) Shannon diversity, and (C) Faith's Phylogenetic Diversity (PD) across all samples, demonstrating sufficient sequencing depth to capture microbial diversity. Dashed vertical line indicates the rarefaction depth of 45,000 sequences per sample used for downstream analyses. Curves were generated by random subsampling of quality-filtered sequences (without replacement) from each sample.


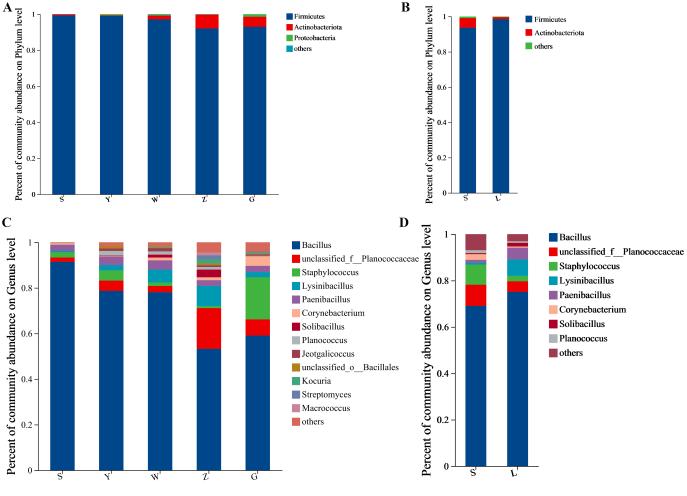


**Figure S2. Dominant bacterial taxa composition**

(A) Phylum-level relative abundance across regions (Small-scale farms). (B) Phylum-level relative abundance across regions (Large-scale farms). (C) Genus-level relative abundance across regions (Small-scale farms). (D) Genus-level relative abundance across regions (Large-scale farms). Only taxa with >1% relative abundance in any sample are shown. Error bars represent standard error of the mean (SEM).


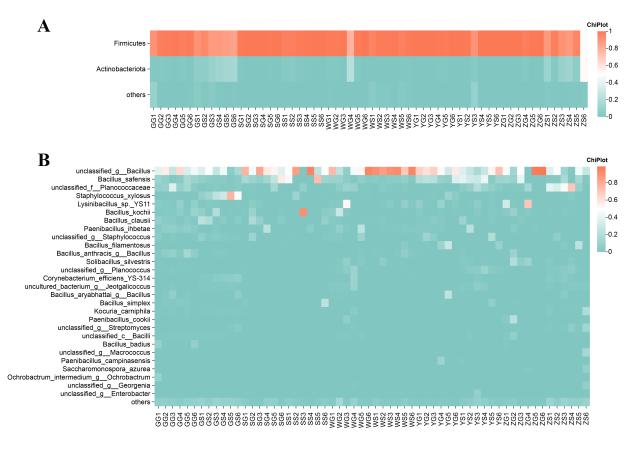


**Figure S3. Heatmap visualization of microbial communities**

1. Phylum and (B) Genus-level relative abundances (log10-transformed) across all samples. Columns represent individual samples grouped by region (color bars) and farming scale (Small-scale/Large-scale). Only taxa present at >0.1% relative abundance in at least 10% of samples are shown.


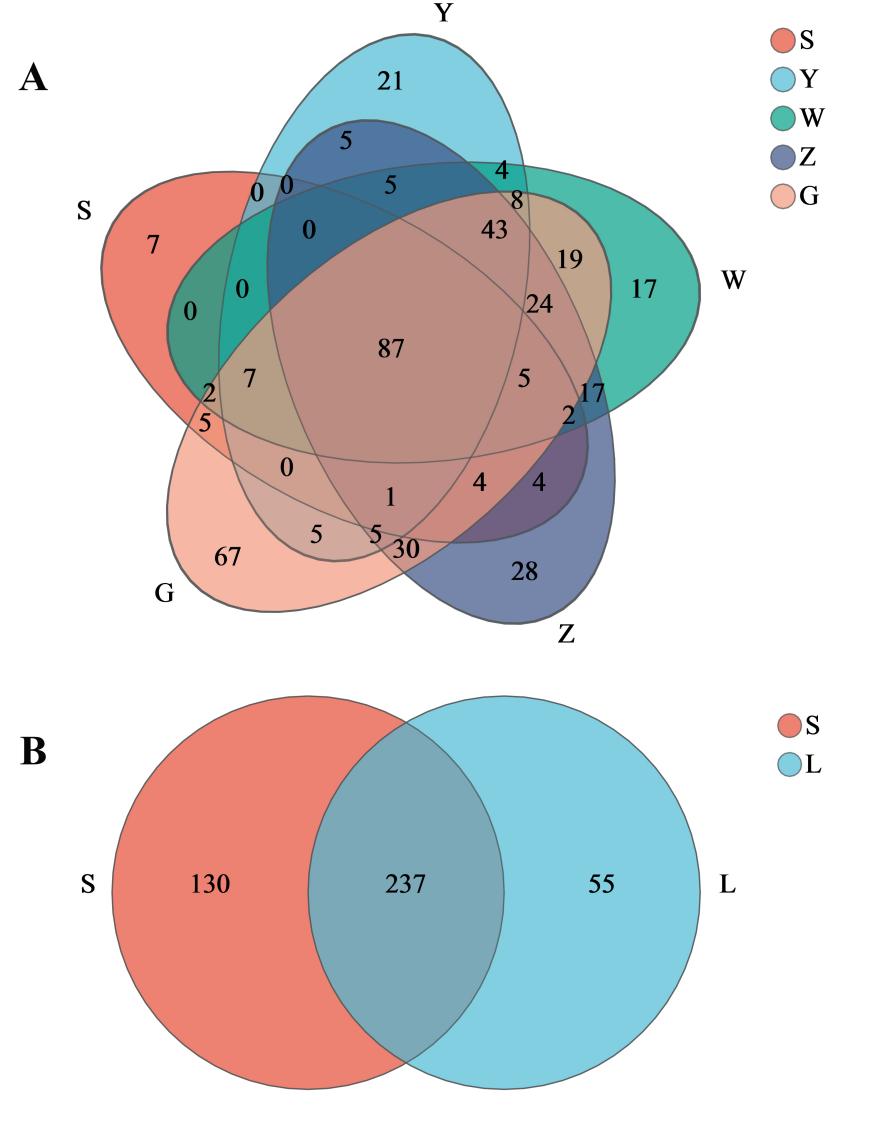


**Figure S4. Shared and unique bacterial genera across groups**

Venn diagrams showing overlap of bacterial genera (rarefied to 45,000 sequences/sample) between (A) regions (core microbiome = 423 shared genera) and (B) farming scales (Small-scale vs Large-scale). Numbers indicate count of unique/shared genera at ≥0.01% relative abundance. Diagrams were generated using rarefied ASV tables to ensure equal sampling depth
